# Comparison of Selenium Source in Preventing Oxidative Stress in Bovine Mammary Epithelial Cells

**DOI:** 10.3390/ani10050842

**Published:** 2020-05-13

**Authors:** Lingling Sun, Fang Wang, Zhaohai Wu, Lu Ma, Craig Baumrucker, Dengpan Bu

**Affiliations:** 1 State Key Laboratory of Animal Nutrition, Institute of Animal Sciences, Chinese Academy of Agricultural Sciences, Beijing 100193, China; sunlingling114289@163.com (L.S.); wangfangrnl@163.com (F.W.); wzh07128@163.com (Z.W.); malu.nmg@163.com (L.M.); 2 Department of Dairy and Animal Science, Penn State University, 324 Henning Bldg., University Park, Jamba, PA 16801, USA; crb@psu.edu; 3 CAAS-ICRAF Joint Lab on Agroforestry and Sustainable Animal Husbandry, Beijing 100193, China; 4 Hunan Co-Innovation Center of Animal Production Safety, CICAPS, Changsha, Hunan 410128, China

**Keywords:** bovine mammary epithelial cell, hydroxy-selenomethionine, antioxidant capacity, oxidative stress

## Abstract

**Simple Summary:**

Selenium (Se) is recognized as an essential trace element in maintaining antioxidant status in humans and animals. Se supplementation in the diets of livestock exist in two forms: Organic and inorganic forms. The organic Se source hydroxy-selenomethionine (HMSeBA) has been proven to be more biologically efficient than inorganic Se to improve antioxidant capacity when fed to dairy cows, since its approval as a feed additive by the European Commission in 2013. However, information on the comparison between HMSeBA and other Se sources in preventing oxidative stress in bovine mammary epithelial cells (BMEC) is limited. The current study compared the effects of HMSeBA, selenomethionine (SeMet) and sodium selenite (SS) on antioxidant capacity and the ability to resist oxidative stress induced by H_2_O_2_ in BMEC. HMSeBA was shown to enhance cellular antioxidant status to resist oxidative damage when compared with SS, but there was no difference between HMSeBA and SeMet. The results of this study provide more information for antioxidant potential of different Se sources in BMEC.

**Abstract:**

Oxidative stress can cause cell damage. Hydroxy-selenomethionine (HMSeBA) is an organic Se source with emerging antioxidant advantages. The objective of this study was to compare the effects of HMSeBA, selenomethionine (SeMet) and sodium selenite (SS) on the antioxidant response and the ability to resist oxidative stress in bovine mammary epithelial cells (BMEC). The BMEC were treated with 0 (Control), 20, 50, 100 and 150 nM HMSeBA, 100 nM SeMet and100 nM SS for 48 h. The results showed that HMSeBA and SeMet treatments had higher glutathione peroxidase (*p* < 0.01) and catalase (*p* = 0.01) activities and mRNA abundance of GPX3 (*p* = 0.02), but lower superoxide dismutase activity compared with SS (*p* = 0.04). The catalase activity (*p* < 0.05) and mRNA abundance of GPX3 (*p* = 0.04) changed in a quadratic manner with the increase of HMSeBA levels. To assess the potential protection of different Se sources against oxidative stress on BMEC, 0 or 50 μM H_2_O_2_ was added to BMEC culture for 3 h after Se pre-treatment for 48 h. The results showed that HMSeBA and SeMet, which did not differ (*p* > 0.05), but further decreased malondialdehyde and reactive oxygen species production compared with SS (*p* < 0.05). In conclusion, HMSeBA showed an enhanced cellular antioxidant status to resist oxidative damage induced by H_2_O_2_ when compared with SS, whereas the effects were similar to SeMet.

## 1. Introduction

Free radicals are produced as by-products during normal metabolic activity, including reactive oxygen species (ROS) and reactive nitrogen species (RNS). During the periparturient and peak lactation periods, dairy cow mammary metabolic changes, associated with parturition and initiation of lactation, are known to increase energy, metabolites or co-factor requirements that produce metabolic stress, thereby leading to an overproduction of free radicals in the mammary gland [1]. Oxidative stress may occur when the production of free radical exceeds the neutralized capacity of the antioxidant system [2], which can potentially cause disease, such as mastitis [3].

Antioxidant defense systems, include antioxidant enzymes and non-enzymatic antioxidants. Selenium (Se) is an essential trace element for animal health [4]. It functions in the antioxidant defense system in the form of selenoproteins, particularly the glutathione peroxidase (GSH-Px) superfamily [5]. Se deficiency and decreased GSH-Px activity are linked to various diseases [6]. The supplement of dietary Se includes both organic (e.g., selenomethionine; SeMet) and inorganic Se (sodium selenite (SS) or sodium selenate). The preferred form of dietary Se for dairy cows is organic (SeMet) because it has been shown that blood GSH-Px activity was 1.4 times higher that of SS, and 1.9 times higher for blood Se concentration [7], which can improve Se status of animals to adapt to different stresses.

The organic Se source hydroxy-selenomethionine (HMSeBA), namely Selisseo, was allowed as a feed additive by the European Commission in 2013 [8]. HMSeBA is synthetic R, S-2-hydroxy-4-methylselenobutanoic acid, known as hydroxy-analog of SeMet, in which the second carbon amino group is substituted by a hydroxyl group. It has been proven to be more biologically efficient than inorganic Se when fed to dairy cows [9,10], finishing pigs [11], broiler chickens [12,13], and laying hens [14]. Furthermore, in vitro studies have shown the role of HMSeBA for selenoprotein synthesis and protection against oxidative stress in intestinal Caco-2 cells [15]. However, the comparison between HMSeBA and other Se sources in preventing oxidative stress in bovine mammary epithelial cells (BMEC) has not been shown. Therefore, the objective of this study was to compare the effect of HMSeBA and its dosage, SeMet and SS on antioxidant response and the ability to resist oxidative stress in BMEC. The results of this study provide more information for antioxidant potential of different Se sources in BMEC.

## 2. Materials and Methods

The experimental protocol was approved by the State Key Laboratory of Animal Nutrition, Institute of Animal Science, Chinese Academy of Agriculture Sciences, Beijing, China (No. IAS20180115).

### 2.1. Cell Culture

The mammary epithelial cell line, used in the current study, was established by Hu et al. using mammary tissue from Chinese Holstein dairy cow, and the details regarding establishment, and purification and chromosomal analysis of BMEC, were published previously [16]. The cells were cultured in 25 cm^2^ culture flasks (HyClone, South Logan, UT, USA) using Dulbecco’s modified Eagle medium/F12 (DMEM/F12) basic medium (Gibco, Carlsbad, CA, USA) containing 10% fetal bovine serum (FBS, Sigma-Aldrich, St Louis, MO, USA) and 1× penicillin/streptomycin (HyClone) at 37 °C in 5% CO_2_. The cells grew to approximately 90% confluence and then were used for subsequent experiments.

### 2.2. Determination of Optimal Culture Time

The cells were seeded at 1 × 10^4^/mL cell density on 96-well plates (180 μL/well, HyClone) and 1 × 10^5^/mL cell density on 6-well plates (2 mL/well, HyClone). After 24 h of incubation in FBS-containing basic medium, the supernatant was replaced with FBS-free DMEM/F12 medium for starved incubation for 8 h, as an adaptation period for subsequent treatments. After adaptation period, cells were washed twice with D-hank’s (balanced salt solution, Sigma-Aldrich), and new FBS-free basic medium containing 0 (Control), 100 nM SS (SS100, Sigma-Aldrich, St Louis, MO, USA), 100 nM SeMet (SeMet100, Sigma-Aldrich) or 100 nM HMSeBA (HMSeBA100, Adisseo Life Science Co., Ltd., Shanghai, China) was added to plate and incubated for 24, 48, and 72 h, respectively. Each experimental treatment had 5 replicates for 96-well plates and 3 replicates for 6-well plates. Experiments were repeated in triplicate unless otherwise stated.

Cell viability analysis: the 3-(4,5-Dimethylthiazol-2-yl)-2,5-diphenyltetrazolium bromide (MTT) kit (Roche, Basel, Switzerland) was used to examine the BMEC survival rate in 96-well plates. Briefly, after each incubation time the medium was replaced with 90 μL fresh basal media and 10 μL MTT and incubated for another 4 h. Subsequently the medium was removed and replaced with 110 μL DMSO, shaken at low speed for 10 min, and quantified with a microplate reader (Infinite F200, Tecan, Switzerland) to determine the absorbance at 490 nm. The cell survival rate of the control group was considered as 1: Cell survival rate = (optical density (OD) value of test group/OD value of control group).

Cellular antioxidant response assay: after each culture time, the culture medium and cells (resuspended with Trypsin/EDTA and washed twice with D-Hank’s, centrifuged at 1000× *g* for 5 min) in 6-well plates were collected for the analysis of GSH-Px, superoxide dismutase (SOD) and catalase (CAT) activities using A005, A001-3 and A007-1 commercial detection kits (Nanjing JianCheng Bioengineering Institute, Nanjing, China) according to the manufacturer’s protocols.

According to the cell survival rate and antioxidant capacity results, the culture time of 48 h was selected as the optimal culture time for subsequent experiments.

### 2.3. Determination of Time and Concentration of H_2_O_2_ Treatment

The cells were seeded on 96-well plates as described above, after adaptation period, fresh FBS-free basic medium containing 0 (Control), 20, 50, 100, 150 μM of H_2_O_2_ (Sigma-Aldrich) was added to culture the BMEC for 1, 3, 5, and 7 h, respectively. The MTT method was used to examine the BMEC survival rate as described above. According to the cell survival rate results, the concentration and culture time of H_2_O_2_ used to induce oxidative stress of BMEC was 50 μM and 3 h.

### 2.4. Cellular Antioxidant Response and Against Oxidative Stress with Different Se Sources

Cells were seeded on 96-well plates and 6-well plates as described above. After adaptation period, new FBS-free basic medium containing 0 (Control), SS100, SeMet100 and 20 nM HMSeBA (HMSeBA20), 50 nM HMSeBA (HMSeBA50), HMSeBA100 and 150 nM HMSeBA (HMSeBA150) was added to culture the BMEC for 48 h. Based on previous studies on Se in BMEC in vitro [17,18], we terminated the supplement dose of Se sources. After 48 h incubation, cells on 96-well plates were measured for survival rate using MTT method. Cells treated in an identical method on 6-well plates were collected for the analysis of antioxidant parameters including GSH-Px, SOD and CAT activities as described above. Cells were also harvested on 6-well plates for the gene expression of *GPX1*, *GPX3*, *SOD1* and *CAT*. Total cellular RNA was isolated using the RNeasy Mini Kit (Qiagen, Valencia, CA, USA). The RNA concentration was determined by ND-1000 spectrophotometer (Nano-Drop Technologies, Wilmington, DE, USA). The electrophoretic analysis of 28S and 18S rRNA subunits was used to assess the RNA integrity. The 100 ng of total RNA of each sample was reversed-transcribed into cDNA using a PrimeScriptTM reverse transcript kit (TaKaRa Biotechnology, Tokyo, Japan). Real time-PCR was used to analyze the gene expression using a SYBR PrimeScriptTM Kit (TaKaRa). Primers for *GPX1*, *GPX3*, *SOD1*, *CAT*, ribosomal protein S9 (RPS9) and ubiquitously expressed transcript isoform 2 (UXT) were designed using Primer Premier and Oligo Software (PREMIER Biosoft International, CA, USA, Table 1). Real time-polymerase chain reactions (PCR) were performed to confirm the specificity of the primers. The specificity of PCR products for different genes were confirmed by gel electrophoresis and sequencing technology. The conditions of Real time-PCR were as follows: 95 °C for 30 s followed by 40 cycles of 5 s at 95 °C then 60 °C for 30 s (Bio-Rad, Hercules, CA, USA). The relative expressions of mRNA were analyzed by the 2^−ΔΔCt^ method using RPS9 and UXT as the internal control gene.

After 48 h Se treatment, the extra 3 plate cells for Se treatment and 6 plate cells for Control on 6-well plates were incubated for another 3 h, and 50 μM H_2_O_2_ was added to Se treatment and positive control groups (3 plates), not for negative control group (0 μM H_2_O_2_, 3 plates). Cells were used for the analysis of malondialdehyde (MDA) concentration and ROS production. Cells were collected with a cell scraper, transferred to a 1.5 mL centrifuge tube, stored at −80 °C for analysis of MDA (A003–4, Nanjing JianCheng Bioengineering Institute). The ROS production was determined by a kit (E004-1-1, Nanjing JianCheng Bioengineering Institute). Briefly, cells were washed twice with D-Hank’s and then incubated with 10 μmol 2′,7′-dichlorofluorescin diacetate (DCFH-DA, as probe) in basal medium at 37 °C for 40 min. A positive control (DCFH-DA with reactive oxygen hydrogen donor) and negative control (basal medium without DCFH-DA) was established. Fluorescence was determined at 500 (excitation) and 525 nm (emission) wavelengths a microplate reader (Infinite F200, Tecan, Switzerland).

### 2.5. Statistical Analysis

The data were statistically analyzed using PROC MIXED procedures of SAS software (version 9.4, SAS Institute Inc., Cary, NC). The treatment, culture time and interaction of treatment × time were as fixed effects in the model, and individual cell culture well was as random effect. Tukey’s test was used for the evaluation of differences between the treatments. Different HMSeBA supplementation levels were detected using linear and quadratic orthogonal polynomial contrasts. *p* < 0.05 were considered significantly different.

## 3. Results

### 3.1. Determination of Optimal Culture Time

**Figure 1:**
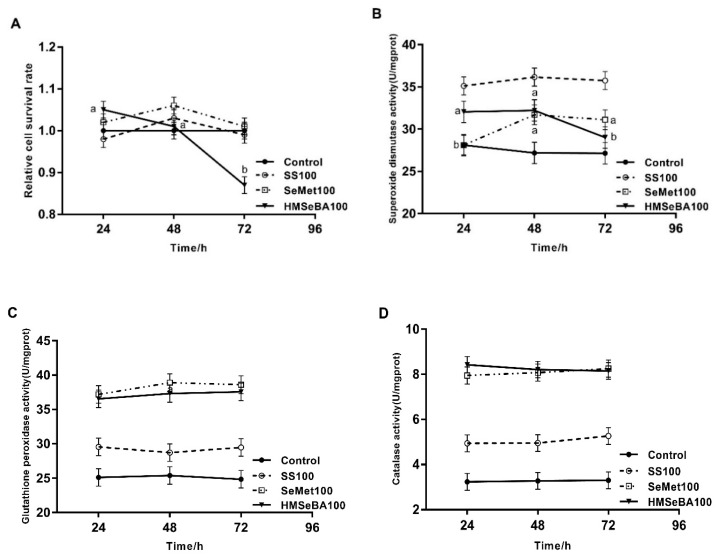
Effect of culture time with different Se sources on cell survival rate (**A**), SOD activity (**B**), GSH-Px activity (**C**) and CAT activity (**D**) of BMEC. All values shown are mean ± SEM from three independent experiments. Different letters in the same Se treatment denote significant differences among culture time.

The effects of culture time on cell survival rate and antioxidant parameters are shown in Figure 1. The cell survival rate in HMSeBA100 treatment decreased significantly at 72 h compared with 24 and 48 h (*p* < 0.05), and there were no significant effects of culture time on cell survival rate in other treatments (*p* > 0.05, Figure 1A). The SOD activity in cells treated with SeMet100 was higher at 48 and 72 h than 24 h (*p* < 0.05), but decreased in HMSeBA100 treatment at 72 h compared with 24 and 48 h (*p* < 0.05, Figure 1B). The activities of GSH-Px and CAT in cells were not affected by culture time in all treatments (*p* > 0.05, Figure 1C and Figure 1D). Based on these results, we selected 48 h as the optimal culture time.

### 3.2. Determination of Time and Concentration of H_2_O_2_ Treatment

The effects of H_2_O_2_ treatments on cell survival rate are shown in Table 2. Different concentrations of H_2_O_2_ were used to incubate BMEC for 1, 3, 5, 7 h inducing 1–6% cell death rate. To produce cell death rate indicated in vivo by Wilde et al. [19], the 50 μM H_2_O_2_ for 3 h (~3% cell death rate) were selected as the conditions to induce oxidative stress in BMEC.

### 3.3. Cellular Antioxidant Response with Different Se Sources

The effects of different Se sources on cell survival rate and antioxidation are shown in Table 3. The cell survival rates were not affected by Se sources and levels of HMSeBA (*p* > 0.05). The activities of GSH-Px, CAT and SOD showed an increase in cells treated with HMSeBA100, SeMet100 and SS100 (*p* < 0.05), compared with the control. Among treatments, the activities of GSH-Px and CAT in cells treated with HMSeBA100 and SeMet100, which did not differ (*p* > 0.05), were greater than SS100 culture (*p* < 0.05), whereas, the SOD activity was lower than SS100 culture (*p* < 0.05). The mRNA abundance of GPX3 in SS100, SeMet100 and HMSeBA100 treatments were 1.1, 1.4 and 1.5 folds compared to control group, and the effect was not similar but not different of GSH-Px activity among Se sources. Se treatments did not influence mRNA abundance of *GPX3*, *SOD1* and *CAT* (*p* > 0.05), and no differences were observed between treatments (*p* > 0.05).

No linear or quadratic effects were detected with increasing HMSeBA levels (*p* > 0.05) for GSH-PX and SOD. Whereas, CAT activity increased in a quadratic manner (*p* = 0.05). The mRNA abundance of *GPX3* increased quadratically with increasing of HMSeBA levels (*p* = 0.04), whereas linear and quadratic effects were not significant for mRNA abundance of GPX1, SOD1 and CAT (*p* > 0.05).

### 3.4. Effects of Different Se Sources on Protection of BMEC Against Oxidative Stress

**Figure 2:**
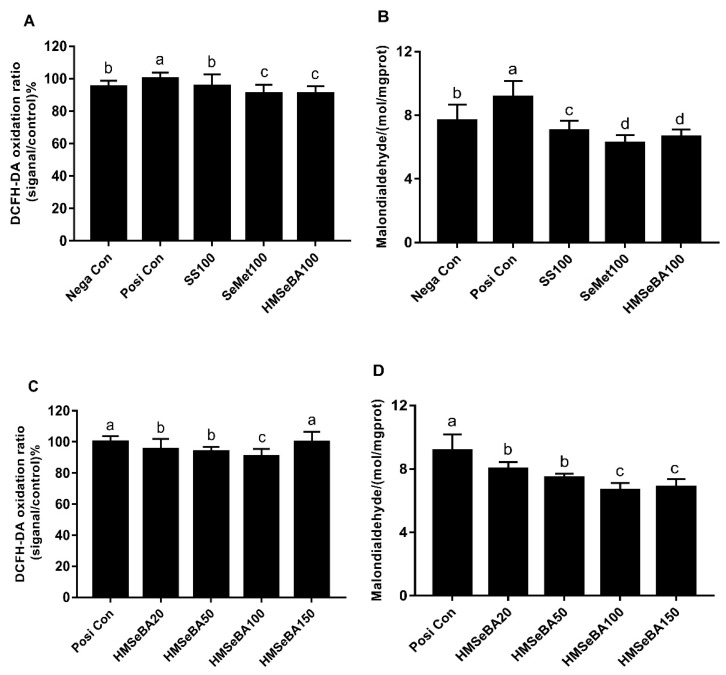
Effect of different Se sources and HMSeBA levels on protection of BMEC against H_2_O_2_ induced oxidative stress. (**A**) DCFH-DA oxidation ratio; (**B**) Malondialdehyde; (**C**) DCFH-DA oxidation; (**D**) Malondiadehyde. Nega Con, negative control (no H_2_O_2_ and no Se); Posi Con, positive control (no Se, H_2_O_2_ treatment); SS100, 100 nM sodium selenite supplementation; SeMet100, 100 nM selenomethionine supplementation; HM20, 20 nM hydroxy-selenomethionine supplementation; HM50, 50 nM hydroxy-selenomethionine supplementation; HM100, 100 nM hydroxy-selenomethionine supplementation; HM150, 150 nM hydroxy-selenomethionine supplementation. All values shown are mean ± SEM from three independent experiments. Different letters denote significant differences between treatments.

The effects of different Se sources on protection of BMEC against oxidative stress are shown in Figure 2. All Se sources-treated cells showed lower ROS production compared with positive control (*p* < 0.05), and among Se sources, HMSeBA and SeMet did not differ, and showed lower ROS production compared with SS and negative control (*p* < 0.05, Figure 2A). The MDA level was greater (*p* < 0.05) in cells treated with H_2_O_2_ (positive control), but all Se sources reduced the level (*p* < 0.05) even lower than untreated cells (negative control). Among treatments, cell treated with HMSeBA and SeMet showed lower MDA levels than SS (*p* < 0.05). No differences in the level of MDA were observed between HMSeBA and SeMet treated cells (*p* > 0.05, Figure 2B).

With the increase of HMSeBA levels, the ROS in cells were decreased (*p* < 0.05) except for HMSeBA150 treated cells (*p* > 0.05), which had the similar value with the positive control culture (Figure 2C). The MDA concentration in cells decreased with the increase of HMSeBA levels (*p* < 0.05), whereas, the level of MDA for cells treated with HMSeBA150 did not show a greater reduction than HMSeBA100 (*p* > 0.05, Figure 2D).

## 4. Discussion

In the current study, we investigated the effects of different Se sources, HMSeBA, SeMet and SS, and dose effect of HMSeBA on cell viability, antioxidant status and resistance to H_2_O_2_- induced oxidative stress in BMEC. HMSeBA as an organic Se source and its effect on BMEC has not been established. Cell survival rate [20], with different Se source treatments, found that no Se treatment reduced cell survival rate and established no differences between treatments. This result showed that incubation with different Se sources and doses for 48 h did not show any cytotoxic effect on BMEC.

Selenium’s nutritional level is closely related to the activity of antioxidant enzymes, such as GSH-Px, SOD and CAT included in the antioxidant system. Selenium is a component of GSH-Px [21], the latter of which is often used as an indicator for evaluating Se status. Gong et al. indicated that dairy cows supplemented with Se yeast, which SeMet is the predominant form of Se [22], had higher GSH-Px activity in the serum compared with SS supplementation [23]. Sun et al. showed that 0.3 mg/kg DM HMSeBA treatment increased serum GSH-Px activity of dairy cows compared with the same dose of SS supplementation [9]. Bansal and Tranum examined Se status changes of yeast cells with inorganic and organic Se supplementation and found that increased GSH-Px activity with SeMet [24]. Consistent with the above finding, we found organic Se can improve GSH-Px activity more efficient than SS with BMEC. However, there was no difference between HMSeBA and SeMet on GSH-Px activity change. This may be due to HMSeBA serving as a precursor of SeMet, being metabolized in the same way as SeMet [25]. We investigated mRNA expression of GPx1 and GPx3, which are the main GPx forms expressed in BMEC [26,27]. The expression of GPx3 is consistent with the activity of GSH-Px, whereas there were no differences of *GPx1* expression between treatments. The expression level of *GPx1* and *GPx3* in BMEC is different while that found in bovine milk has *GPx3* levels expressed 100 times greater than that of *GPx1* [27,28]. With the increased dose of HMSeBA, the expression of *GPx3* changed quadratically. It may be that the greater dose of HMSeBA inhibited the gene expression of *GPx3*, but GSH-Px activity did not show a similar trend. It may also be that the GSH-Px activity is the combined effect of *GPx1* and *GPx3* expression. SOD catalyzes the transformation of superoxide anions into H_2_O_2_ in the mitochondria: the first step in ROS neutralization [29]. In the current study, SS increased SOD activity greater than that of HMSeBA and SeMet. Jamwal and Niyogi evaluated the effects of SS and SeMet against arsenite cytotoxicity using primary rainbow trout hepatocytes and they revealed that SS ameliorated oxidative stress by augmenting SOD, whereas SeMet mechanism was different [30]. CAT can decompose H_2_O_2_ to H_2_O and O_2_ (catalytic activity), which is also an antioxidant enzyme to evaluate the Se status [31]. CAT had a similar trend with GSH-Px activity among Se sources and reflected the better effect of organic Se to improve the antioxidant capacity. Surprisingly, *SOD* and *CAT* gene expressions were not affected by Se treatments, indicating Se may not change the enzyme activities by changing their mass.

To measure the potential protection of different Se sources against oxidative stress, we measured the levels of ROS and MDA in BMEC, which were pretreated with corresponding Se sources and doses after a challenge of 0 or 50 μM H_2_O_2_. The ROS are considered as cellular damaging agents historically, however, appropriate level of ROS can be involved in cellular signaling process to activate antioxidant enzyme expression, in order to maintain the balance of antioxidant system [32]. When the ROS production overwhelms the capacity of these systems, oxidative stress develops. In the current study, Se supplementation decreased the ROS production, induced by H_2_O_2_ compared with control. HMSeBA and SeMet culture were more efficient than SS and is likely due to HMSeBA and SeMet treatments inducing higher antioxidant enzyme activities. However, HMSeBA150 treatment increased ROS level to that of the positive control, and it may be due to the Se supplementation being too high to induce oxidative stress. MDA is one of the final products of lipid peroxidation in the cells. An increase in free radicals causes overproduction of MDA, which is commonly utilized as a marker of oxidative stress and the antioxidant status [33]. In the current study, all Se supplementation decreased the cellular MDA concentration compared with the positive control. This indicated a protective effect of Se against cell lipoperoxidation and further showed the increase in antioxidant capacity with Se supplementation. HMSeBA and SeMet resulted in better protective effects than SS, whereas high dose (150 nM) of HMSeBA did not show greater effects.

## 5. Conclusions

In conclusion, we found that HMSeBA and SeMet increased antioxidant capacity of BMEC compared with SS, therefore possibly more beneficial to protect BMEC against oxidative stress, reflected in decreased ROS and MDA content. There were no differences between HMSeBA and SeMet in improving antioxidant capacity of BMEC, and the effect of HMSeBA treatment was dose-dependent.

## Figures and Tables

**Table 1:** Primer sequences used for Real-time PCR.

Genes ^1^	Accession Number	Primer Sequences
*GPX1*	NM_174076.3	F: CGCTGGTCCTATCCATCCC R: GCTCACATCTGGCACTTTATTC
*GPX3*	NM_174077.5	F: GCTGGCAAATACATCCTCTT R: GGGAAGCCCAGAATGACC
*SOD1*	NM_174615.2	F: AAACCAGATGACTTGGGCAGAG R: AGGCCAAACGGCTTCCAG
*CAT*	NM_001035386.2	F: ACGGCGACTATCCTCTTA R: AAGCCAACTGTTCAACCT
*RPS9*	NM_001101152.2	F: ATCCCGTCCTTCATCGTGC R: CCCTTCTTGGCGTTCTTCC
*UXT*	NM_001037471.2	F: TGGACCATCGTGACAAGGTA R: TGAAGTGTCTGGGACCACTG

^1^*GPX1*, glutathione peroxidase 1; *GPX3*, glutathione peroxidase 3; *SOD1*, superoxide dismutase 1; *CAT*, catalase; *RPS9*, ribosomal protein S9; *UXT*, ubiquitously expressed transcript isoform 2.

**Table 2:** The effects of H_2_O_2_ treatments on cell survival rate.

Concentration of H_2_O_2_ (μM)	Culture Time (h)				SEM
	1	3	5	7	
0	1.00	1.00	1.00	1.00	0.02
10	0.99	0.99	0.99	0.98	0.02
30	0.99	0.99	0.98	0.98	0.03
50	0.98	0.97	0.96	0.96	0.02
100	0.96	0.95	0.95	0.94	0.02

The survival rate of 0 (Control) group was considered as 1, and the cell survival rates of other groups were relative to control group. All values shown are mean ± SEM from three independent experiments.

**Table 3:** Effect of different Se sources on cell survival rate and antioxidation with BMEC.

Items	Treatment ^1^							SEM	*p*-Value			
	Control	SS100	SeMet100	HM20	HM50	HM100	HM150		Trt ^2^	Source ^3^	HMSeBA level ^4^	
											Linear	Quadratic
Cell survival rate	1	0.99	0.97	0.97	0.97	1.01	0.99	0.02	0.11	0.31	0.18	0.42
Antioxidant enzymes activity, U/mgprot												
Glutathione peroxidase	28.0 ^c^	32.9 ^b^	37.2 ^a^	32.8	33.4	36.5 ^a^	36.9	1.2	0.05	<0.01	0.17	0.1
Catalase	3.26 ^c^	4.66 ^b^	7.55 ^a^	3.54	6.21	6.96 ^a^	7.31	0.36	<0.01	0.01	0.42	0.05
Superoxide dismutase	27.9 ^c^	35.2 ^a^	30.2 ^b^	30.7	32.1	31.6 ^b^	32.6	1.4	<0.01	0.04	0.4	0.23
Gene expression fold change relative to control												
Glutathione peroxidase 1	1	1.2	1.2	1.1	1.2	1.2	1.2	0.1	0.43	0.24	0.48	0.69
Glutathione peroxidase 3	1 ^b^	1.1 ^b^	1.4 ^a^	1.2	1.2	1.5 ^a^	1.3	0.1	<0.01	0.02	0.76	0.04
Catalase	1	1	0.9	1.1	1.1	1	1	0.1	0.36	0.54	0.41	0.35
Superoxide dismutase 1	1	1.1	1.2	1.2	1	1.1	1.1	0.1	0.24	0.43	0.77	0.61

^1^ SS100, 100 nM sodium selenite supplementation; SeMet100, 100 nM selenomethionine supplementation; HM20, 20 nM hydroxy-selenomethionine supplementation; HM50, 50 nM hydroxy-selenomethionine supplementation; HM100, 100 nM hydroxy-selenomethionine supplementation; HM150, 150 nM hydroxy-selenomethionine supplementation; ^2^ Trt, treatment: includes Control, SS100, SeMet100, HM20, HM50, HM100 and HM150; ^3^ Source: a comparison of SS100, SeMet100 and HM100; ^4^ Linear and quadratic analysis for Control, HM20, HM50, HM100 and HM150. All values shown are mean±SEM from three independent experiments. ^a,b^ Different letters denote significant differences among Se sources with the same dose.
